# Pseudoxanthoma elasticum-like papillary dermal elastolysis in non-exposed skin^☆^^☆☆^

**DOI:** 10.1016/j.abd.2019.08.024

**Published:** 2020-02-12

**Authors:** Nuria Setó-Torrent, Maribel Iglesias-Sancho, Jorge Arandes-Marcocci, María Teresa Fernández-Figueras

**Affiliations:** a Department of Dermatology, Hospital Universitari Sagrat Cor-Grupo Quirón Salud, Barcelona, Spain; b Department of Pathology, Hospital Universitari Sagrat Cor-Grupo Quirón Salud, Barcelona, Spain

**Keywords:** Dermis, Elastic tissue, Female, Pseudoxanthoma elasticum

## Abstract

Pseudoxanthoma elasticum-like papillary dermal elastolysis is an acquired elastic tissue disorder clinically similar to pseudoxanthoma elasticum in the absence of systemic involvement. Histopathologically, special staining of elastic fibers demonstrates a total or partial band-like loss of elastic fibers in the papillary dermis. Although ultraviolet radiation seems to be one of the main etiological factors in this entity, we report a case of pseudoxanthoma elasticum-like papillary dermal elastolysis on the neck of a woman who wore hijab.

A 54-year-old female of Moroccan origin who habitually wears a hijab presented a 2 year history of mildly pruritic lesions on the neck. She denied systemic symptoms and family history of similar findings. Her medical history included mixed anxiety-depressive disorder treated with olanzapine and sertraline. Physical examination revealed white-to-yellowish millimetric non-follicular papules on the lateral aspects of the neck and supraclavicular fossae (Fig. 1). Dermoscopic examination showed multiple white-colored non-follicular papules, coalescing into plaques with arboriform vessels (Fig. 2). The biopsy showed slight sclerosis of the papillary dermis with neovascularization and a mild inflammatory infiltrate including lymphocytes and some melanophages (Fig. 3). In the same area, van Gieson stain demonstrated a decrease in the number of elastic fibers that were often thin and fragmented (Fig. 4) compatible with pseudoxanthoma elasticum-like papillary dermal elastolysis (PXE-PDE). Cardiac and ophthalmological investigations performed were unremarkable.

**Figure 1:**
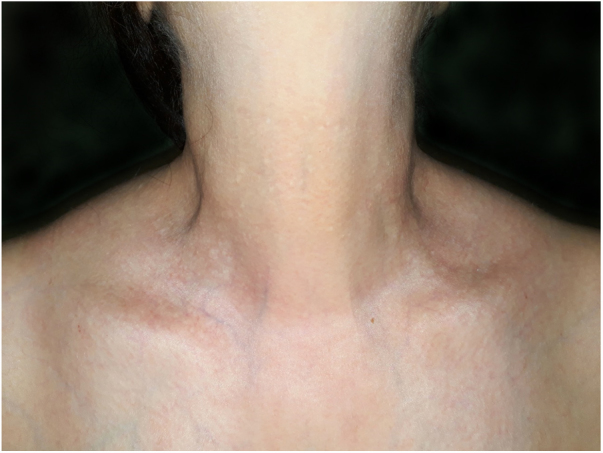
Whitish papules on the neck and supraclavicular fossae.

**Figure 2:**
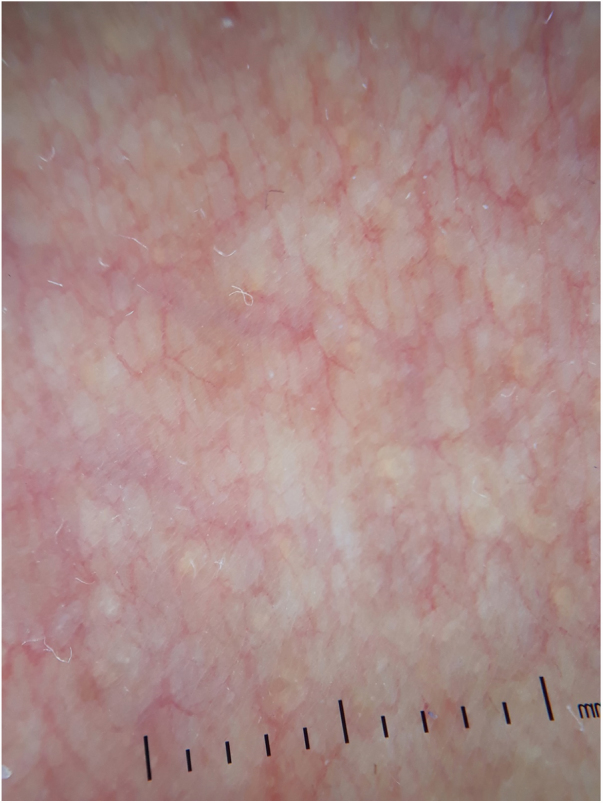
Multiple whitish non-follicular papules, coalescing into plaques with linear vessels, on dermoscopy.

**Figure 3:**
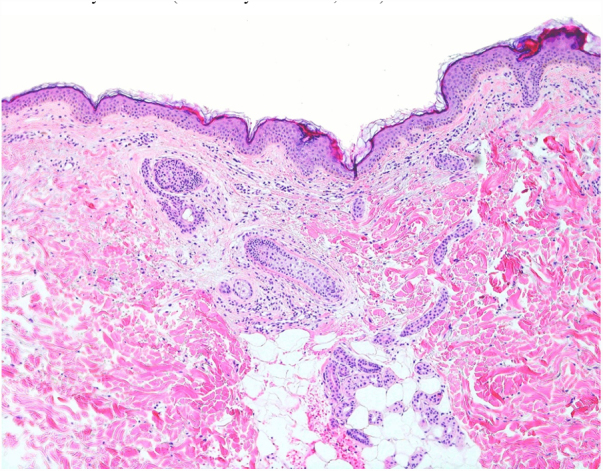
Slight sclerosis of the papillary dermis, neovascularization and a mild inflammatory infiltrate (Hematoxylin & eosin, x100).

**Figure 4:**
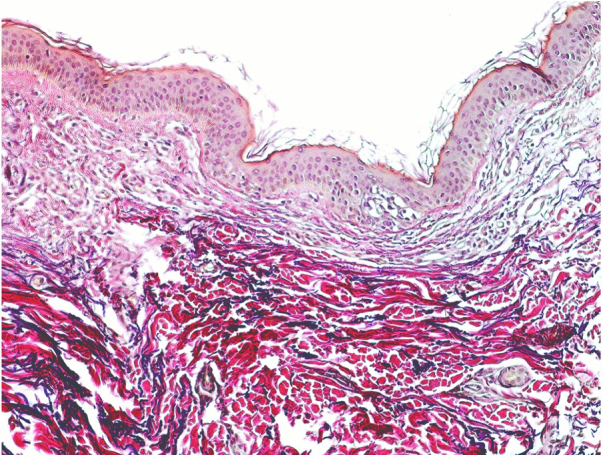
Reduction of elastic fibers in the papillary dermis (van Gieson, x200).

PXE-PDE is a rare acquired elastic tissue disorder characterized by non-follicular yellowish papules coalescing into plaques with predilection for neck, supraclavicular fossae and flexural areas.^1^ The lesions are usually asymptomatic, but mild itch is sometimes reported, as seen in our case. To date, it affects exclusively women mostly in middle age^2^ and it is not associated with any systemic involvement. Dermoscopic findings consist of multiple white-colored non-follicular papules, coalescing into plaques with linear vessels.^3^

Histopathologically, hematoxylin eosin staining does not reveal any specific changes. The focal inflammatory changes present in our case have not been described previously; however, it is presumed that elastic fiber loss could be the result of a transient phenomenon of inflammation. Special staining of elastic fibers with van Gieson or orcein stains are required to demonstrate a total or partial band-like loss of elastic fibers in the papillary dermis.^2^ Calcification or fragmentation of the elastic fibers is absent. Immunohistochemical studies using monoclonal antibodies against antibody P component can also demonstrate partial or complete loss of elastic fibers in papillary dermis.^1^ The presence of melanophages in the papillary dermis constitutes an additional helpful diagnostic feature.^4^

The cause of PXE-PDE remains unclear, and some etiopathogenic theories have been proposed: ultraviolet radiation, intrinsic aging, abnormal elastogenesis, and genetic or inheritable factors.^1^, ^2^ In our case, ultraviolet radiation's etiopathogenic theory is unlikely because the patient wore hijab.

Differential diagnosis of PXE-PDE includes white fibrous papulosis of the neck, mid-dermal elastolysis, and papillary dermal elastosis. Nevertheless, the main differential diagnosis must be established with pseudoxanthoma elasticum (PXE), a hereditary disorder caused by mutation on ABCC6 gene. Clinically, PXE resembles PXE-PDE, but it appears at a younger age, and it is usually associated with ocular and cardiovascular complications. Histopathologically, PXE presents fragmentation and calcification of elastic fibers demonstrated with von Kossa stain.

Treatments for PXE-PDE, including topical retinoids, have shown poor results^2^; however, non-ablative fractional resurfacing laser has demonstrated to be effective in some cases.^5^

Herein we present a case of PXE-PDE in a patient who did not receive UV radiation because she wore hijab. In our opinion, more studies are needed in order to better understand the etiopathogenesis of PXE-PDE. It is important that dermatologists recognize this entity and differentiate it from PXE to avoid unnecessary investigation. Clinicopathologic correlation is important and elastic tissue stains are required to correctly diagnose PXE-PDE.

## Financial support

None declared.

## Authors’ contributions

Nuria Setó Torrent: Approval of the final version of the manuscript; elaboration and writing of the manuscript; intellectual participation in the propaedeutic and/or therapeutic conduct of the studied cases; critical review of the literature; critical review of the manuscript.

Maribel Iglesias Sancho: Approval of the final version of the manuscript; critical review of the manuscript.

Jorge Arandes Marcocci: Approval of the final version of the manuscript; critical review of the manuscript.

María Teresa Fernández Figueras: Approval of the final version of the manuscript; critical review of the manuscript.

## Conflicts of interest

None declared.
